# Genetic variation and expression diversity between grain and sweet sorghum lines

**DOI:** 10.1186/1471-2164-14-18

**Published:** 2013-01-16

**Authors:** Shu-Ye Jiang, Zhigang Ma, Jeevanandam Vanitha, Srinivasan Ramachandran

**Affiliations:** 1 Temasek Life Sciences Laboratory, 1 Research Link, the National University of Singapore, Singapore, 117604, Singapore

## Abstract

**Background:**

Biological scientists have long sought after understanding how genes and their structural/functional changes contribute to morphological diversity. Though both grain (BT×623) and sweet (Keller) sorghum lines originated from the same species *Sorghum bicolor* L., they exhibit obvious phenotypic variations. However, the genome re-sequencing data revealed that they exhibited limited functional diversity in their encoding genes in a genome-wide level. The result raises the question how the obvious morphological variations between grain and sweet sorghum occurred in a relatively short evolutionary or domesticated period.

**Results:**

We implemented an integrative approach by using computational and experimental analyses to provide a detail insight into phenotypic, genetic variation and expression diversity between BT×623 and Keller lines. We have investigated genome-wide expression divergence between BT×623 and Keller under normal and sucrose treatment. Through the data analysis, we detected more than 3,000 differentially expressed genes between these two varieties. Such expression divergence was partially contributed by differential *cis*-regulatory elements or DNA methylation, which was genetically determined by functionally divergent genes between these two varieties. Both tandem and segmental duplication played important roles in the genome evolution and expression divergence.

**Conclusion:**

Substantial differences in gene expression patterns between these two varieties have been observed. Such an expression divergence is genetically determined by the divergence in genome level.

## Background

Grain sorghum is the fifth most important cereal crop, providing food, feed and fiber for the world. Sweet sorghum has been evaluated as a viable feed stock for bio-ethanol production due to its high biomass yield and sugar content. Both grain and sweet sorghum lines originated from the same species “*Sorghum bicolor* L.”. These lines were found in sorghum landraces but modern sorghum cultivars were domesticated through breeding programs. In addition, they exhibit considerable differences in their phenotype. The grain sorghum genome AT×623 was first sequenced by methylation filtration technology [1]. On the other hand, the BT×623 genome was then completely sequenced by the short-gun sequencing technique and total of 36,338 loci were annotated with protein-coding transcripts [2]. Recently, Zheng et al. (2011) have re-sequenced two sweet sorghum and one additional grain sorghum genomes and identified a large numbers of SNPs (Single Nucleotide Polymorphisms), Indels (Insertion and Deletion), PAVs (presence/absence variations) and CNVs (copy number variations) [3]. However, our detailed analysis revealed that the differentiation in gene functions between the grain (BT×623) and sweet (Keller) sorghum lines might not directly contribute to their phenotypic divergence (see below), which raises the question how the obvious morphological variations between these sorghum lines occurred in a relatively short evolutionary or domesticated period.

Accumulated data demonstrated that expression divergence correlated with phenotype variations and manipulations of appropriate gene expression are sufficient for recreating phenotypic differences [4,5]. Expression profiling is one of the most important tools for dissecting biological functions of genes. However, no commercial microarray chips are available for sorghum to analyze the expression profiles. The first sorghum cDNA microarray chips were developed to identify differentially expressed genes under various treatments [6,7] but were not commercially available. The chips were used for detecting the expression of only 12,982 unique genes. Calviño et al. (2008; 2009) identified 154 differentially expressed genes between grain and sweet sorghum lines by using an Affymetrix sugarcane genechip [8,9]. However, only conserved sorghum genes could be detectable using the sugarcane chip. To our knowledge, no other microarray-based genome-wide expression analysis has been carried out in sorghum species. In this study, we first designed a sorghum custom microarray chip based on the genome annotation and available expressed sequence tag (EST) datasets. The chip comprises of 41,905 probes, representing 35,465 annotated loci and 6,440 sorghum ESTs, which have not been mapped on the annotated loci. Subsequently we analyzed and compared the transcription profiles between the grain and sweet sorghum lines. Such analysis of our data revealed around 30,000 expressed genes in both lines. These two lines showed difference in their transcriptomes with considerable numbers of variety-specifically or differentially expressed genes and they also exhibited expression difference in response to sucrose treatment.

Another raised question is about the molecular basis of expression divergence. Similar to other genomes, gene duplication and expansion were also observed in the sorghum genome [2]. Expression divergence of duplicated/expanded genes is a subject of great interest to geneticists and evolutionary biologists because it may contribute to the retention and functional divergence of duplicated/expanded genes [10]. Meagher (2010) proposed the hypothesis that epitype and associated phenotypes evolved by gene duplication, divergence, and subfunctionalization [11]. To investigate the contribution of gene duplication and expansion to expression divergence, we have identified tandemly or segmentally duplicated as well as expanded sorghum genes by mobile elements in a genome-wide level and subsequently investigated their expression divergence under normal growth conditions and sucrose treatments. Our data showed that higher expression divergence was observed in segmentally duplicated genes when compared to the tandemly duplicated genes. These duplicated genes in the grain sorghum experienced higher ratio of expression divergence when compared with those in the sweet sorghum. Limited expression divergence was observed for those genes expanded by mobile elements between BT×623 and Keller.

Finally, although expression divergence has been observed in several closely related species [12-14], little is known about the mechanisms underlying this divergence. To figure out the mechanisms underlying these expression variations among orthologous or paralogous genes, we further analyzed the regulatory motifs of their promoter regions. Our data showed that SNPs or structural variations (SVs) have significantly contributed to the expression divergence. In addition, DNA methylation may also play an important role in the species divergence through expression regulation of genes.

## Results

### Phenotypic and genetic variations between the grain and sweet sorghum

**Figure 1:**
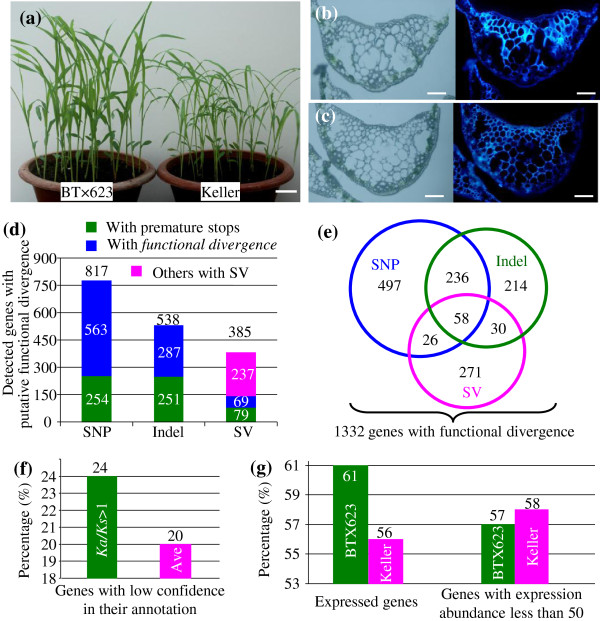
**Phenotypic and genotypic variations between BT×623 and Keller. (a)** Comparison between BT×623 and Keller at seedling stage. Bar in **(a)** is 3 cm. **(b)** and **(c)** Cross-sections of BT×623 and Keller leaves at the mid-rib regions under light microscopy (left) and under UV light with epifluorescence (right), respectively. Bars in **(b)** and **(c)** are 150 μM. **(d)** Detected genes with putative functional divergence with either premature stops or *Ka/Ks*>1. **(e)** Summary of genes with functional divergence. The venn diagram shows the relationship of divergent genes in their variation types. The blue, green and pink circles indicate the genes with variations from SNP, Indel and SV, respectively. **(f)** Percentage of genes with *Ka/Ks*>1 and with low confidence in their annotation. **(g)** Analysis of expression abundance of genes with functional divergence.

Both the grain (BT×623) and sweet (Keller) sorghum varieties showed obvious difference in their visible morphological characteristics. For example, at the seedling stage, BT×623 grew stronger than Keller (Figure 1a). Keller contained higher percentage of stem sugar content, and its brix degree is around 16.7%, statistically higher than 10.3% in BT×623 (P<0.01). Both varieties also showed the difference in the developmental stage of tracheary elements during xylem formation (Figure 1b and c). The difference was observed by epifluorescence, which was emitted from dead cells. It was obvious that BT×623 developed these elements earlier with stronger fluorescence than Keller at the similar stage.

Since there are obvious phenotypic variations between these two lines, we expect a significant difference in their genotypes. The re-sequencing results revealed up to 85,041 SNPs, 16,781 Indels and 1,847 SVs in all annotated gene regions, accounting for 20%, 34% and 27% of total SNPs, Indels and SVs, respectively [3]. These variations cover 14,782 genes for SNPs, 7,977 genes for Indels and 2,071 genes for SVs. However, most of these variations may not affect functions of these genes. We have detected only 254, 251 and 79 genes suffered from SNP, Indel and SV, respectively, which may encode truncated proteins due to premature stops in Keller genes (Figure 1d). We subjected the remaining genes to the *Ka/Ks* analysis (where *Ka* = nonsynonymous substitutions per site, and *Ks* = synonymous substitutions per site) and C-value test (see methods). We have detected 563, 287 and 69 genes from SNP, Indel and SV, respectively, with functional divergence (Figure 1d). However, for structural variations, up to 237 genes were not investigated in their substitution rates since they were from deletion or copy number variation. Totally, 817, 538 and 385 genes have been detected with functional or protein divergence between BT×623 and Keller varidue to SNP, Indel and SV, respectively. Some of these genes have been undergone more than one type of variations. For example, 58 genes were detected with variations from SNP, Indel and SV (Figure 1e). Thus, a total of 1,332 genes were identified with functional or protein divergence between BT×623 and Keller. Further analysis showed that higher percentage of genes with *Ka/Ks* > 1 (24%) were annotated with low confidence while a total of 5197 genes were annotated in this class (20%) in the genome-wide level (Figure 1f). Furthermore, only 61% and 56% of these genes showed expression in our microarray analysis in BT×623 and Keller, respectively (Figure 1g). Among the expressed genes, up to 57% in BT×623 and 58% in Keller were identified with expression abundance less than 50 (Figure 1g). All these data suggested that in our analysis some of these genes with functional or protein divergence might have evolved into pseudogenes. Therefore, although significant difference in their visible phenotype between grain and sweet sorghum, relatively less divergence was observed in their encoded gene level.

### Contribution of gene duplication and mobile elements to genome divergence within and between BT×623 and Keller

**Figure 2:**
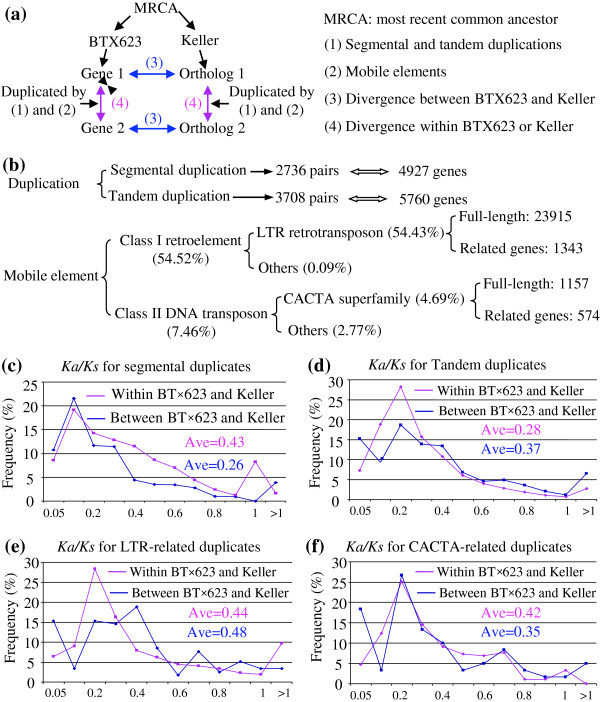
**Contribution of duplication and mobile elements to genome divergence. (a)** A simple model to show the contribution of duplication and mobile elements to gene divergence. **(b)** Genome-wide identification of duplication and mobile elements. Both tandem and segmental duplication related genes have been identified in genome-wide level. For mobile elements, both LTR retrotransposons and CACTA DNA transposons have been selected for genome-wide identification since these two elements consist of more 95% of mobile elements. **(c)** to **(f)***Ka/Ks* analysis of duplicated pairs from segmental and tandem duplication, LTR-retrotransposition, CACTA transposition, respectively. Red and blue lines show the *Ka/Ks* values from within and between BT×623 and Keller, respectively.

Re-sequencing results showed that genome-wide duplication in either BT×623 or Keller might not occur after divergence from the most recent common ancestor (MRCA) of these two varieties. The data also showed that majority of segmental and tandem duplication as well as transposition of mobile elements occurred in their MRCA. To explore the contribution of segmental/tandem duplication and mobile elements to genome divergence within and between these two species (Figure 2a), we have genome-widely identified these duplicated genes or mobile element related genes. We have identified 4,927 segmentally duplicated genes from 2,739 segmental pairs (Figure 2b). We have also identified 3,708 tandem pairs consisting of 5,760 tandemly duplicate genes (see Methods). On the other hand, more than 60% of the sorghum genome was identified to be class I retroelements (54.52%) or class II DNA transposon (7.46%) (Figure 2b; [2]). Among the class I retroelements, majority of them is LTR retrotransposon, consisting of 54.43% of the genome. We have identified total of 23,915 full-length of LTR retrotransposons and of which only 1,343 genes were annotated (Figure 2b). For the class II DNA transposons, the largest class is the CACTA superfamily, accounting for 4.69% of the genome. We identified a total of 1,157 full-length CACTA elements with 574 captured genes (Figure 2b). To explore the functional or protein divergence of these genes, we subjected their sequences to *Ka/Ks* analysis (Figure 2c-f). Generally, majority of these genes (~90%) were subjected to purifying selection with *Ka/Ks* less than 1 either within or between the two varieties. For segmental duplicates, the average *Ka/Ks* value was 0.26 between BT×623 and Keller while the value was up to 0.43 within these two genomes (Figure 2c). The *Ka/Ks* value is 0.28 and 0.34, respectively for tandem pairs (Figure 2d). These data suggested that, after segmental/tandem duplication in their MRCA, lower divergence might have occurred between genes and their orthologs. Similar result was also observed for CACTA- or LTR-related duplicates (Figure 2f and e).

### Custom microarray analysis revealed variety-specifically or differentially expressed genes between BT×623 and Keller

**Figure 3:**
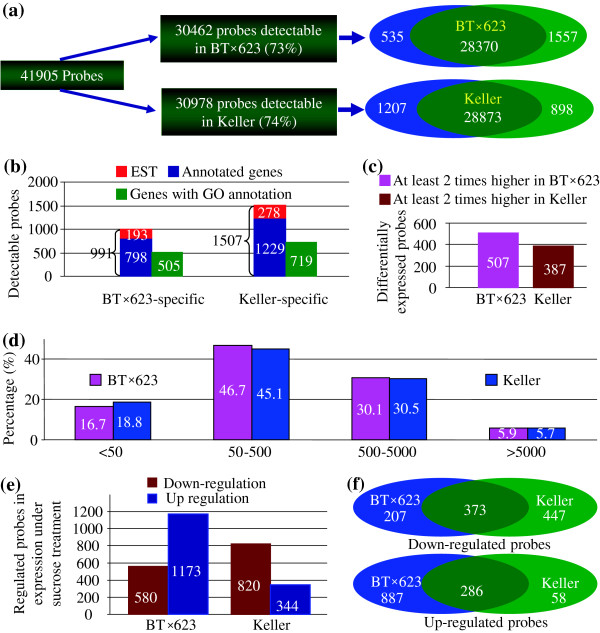
**Expression divergence of genes between BT×623 and Keller. (a)** General expression profile of genes in both BT×623 and Keller under normal and sucrose treatments. **(b)** Detection of genes with BT×623- or Keller-specific expression. **(c)** Identification of genes with differential expression abundance between BT×623 and Keller. **(d)** General profile of expression abundance in both BT×623 and Keller. **(e)** Genome-wide identification of genes regulated by sucrose treatments. **(f)** A venn diagram showing the distribution of regulated genes in either BT×623 or Keller.

We have identified 30,462 probes with expression signals in BT×623. Among them, 1,557 probes were detected only in sucrose-treated tissue samples, 535 only in normal growth conditions and the remaining 28,370 were expressed in both normal and sucrose-treated tissues (Figure 3a). In Keller, total of 30,978 probes were detectable with 1,207 probes in normal growth conditions, 898 in sucrose-treated tissues and 28,873 in both conditions (Figure 3a). Based on the Agilent earray signal detection system, we have detected 991 probes with expression only in BT×623 but not in Keller and these probes were from 193 ESTs and 798 annotated genes, respectively; similarly, we have also detected 1,507 Keller-specific probes including 278 from ESTs and 1,229 from annotated genes (Figure 3b). Our data showed that some of genes exhibited significant difference in their expression abundance between BT×623 and Keller (Figure 3c). We have detected total of 507 genes with at least twice higher in their expression level in BT×623 when compared with Keller. In Keller, total of 387 genes were observed with at least twice higher in their expression signal when compared with BT×623. We classified all expressed genes into 4 groups based on their expression level (Figure 3d). Genes with low abundance (the processed signal is less than 50) account for 16.7% and 18.8% of total expressed genes in BT×623 and Keller, respectively. More than 45% of genes were expressed with middle abundance (50–500) and around 30% of genes were with high abundance (500–5,000). Only less than 6% of genes exhibited very high expression level (>5,000). Our data showed that similar percentages of genes have been detected between BT×623 and Keller in each group, which was not statistically different (Z-test, P>0.05).

To explore why both BT×623 and Keller show the obvious difference in their brix degree, we carried out the comparative expression analysis under sucrose treatment. In BT×623, only 580 down-regulated genes were identified whereas up to 1,173 genes were detected with up-regulated expression patterns (Figure 3e). However, in Keller, total of 820 genes were down-regulated and only 344 genes were regarded as up-regulation in their expression (Figure 3e). Thus, our data revealed that the number of up-regulated genes in BT×623 was at least twice as many as the down-regulated genes; and in Keller, contradictory situation was observed, that was, up-regulated genes were only half of the down-regulated genes. For both down- and up-regulated genes, considerable numbers of genes were commonly regulated by sucrose treatment in both the grain and sweet sorghum (Figure 3f). Besides the commonly down-regulated genes, up to 447 genes were down-regulated only in Keller and 207 genes were only in BT×623. On the contrary, apart from the commonly up-regulated genes, majority of up-regulated genes were detected only in BT×623 and only 58 up-regulated genes were presented in Keller. These data revealed the obvious difference between the grain and sweet sorghum varieties in their sucrose regulation pathways.

Among the differentially expressed genes under sucrose treatment, we were interested in those genes encoding transcription factors (TFs) or carbohydrate metabolism related genes. Among around 2,000 genes encoding transcription factors, we have identified 88 down-regulated and 38 up-regulated genes (Additional file 1a and b). They are from different families of TFs and both grain and sweet sorghum showed the difference in the sucrose regulation of TFs. Among around 300 carbohydrate metabolism related genes, only 10 of them were down-regulated and 11 were up-regulated (Additional file 1c and d). These results showed that most of these genes were not regulated by sucrose treatment although they were involved in sucrose metabolism.

### Functional annotation of differentially expressed genes between BT×623 and Keller

**Figure 4:**
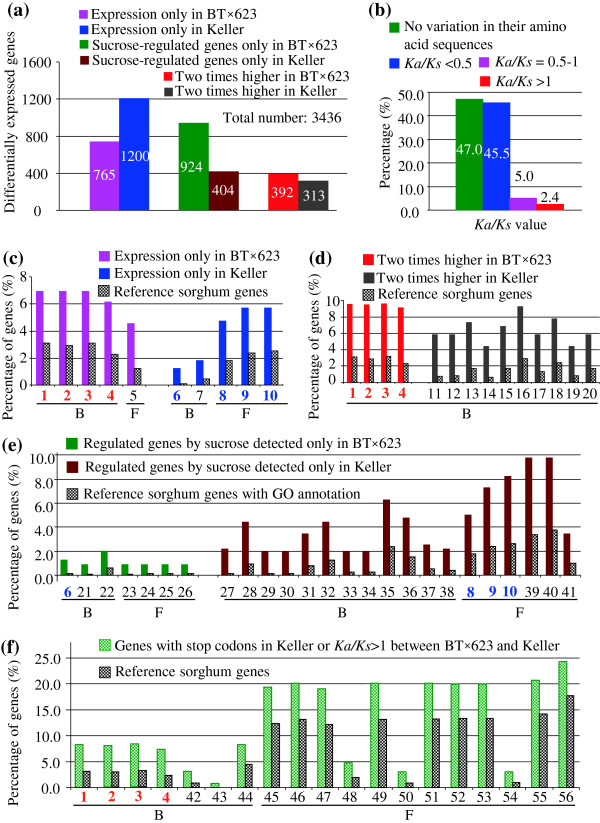
**Functional annotation of differentially expressed genes between BT×623 and Keller.** (**a**) A summary of 6 types of differentially expressed genes between BT×623 and Keller. (**b**) Ka/Ks analysis of differentially expressed genes. (**c**) to (**f**) Functional annotation of over-represented genes. 1, death; 2, programmed cell death; 3, cell death; 4, Apoptosis; 5, helicase; 6, reproductive cellular process; 7, post-embryonic morphogenesis; 8, monooxygenase activity; 9, heme binding; 10, tetrapyrrole binding; 11, flavonoid biosynthesis; 12, flavonoid metabolism; 13, aromatic compound biosynthesis; 14, pigment biosynthesis; 15, cellular amino acid derivative biosynthesis; 16, cellular aromatic compound metabolism; 17, phenylpropanoid biosynthesis; 18, cellular amino acid derivative metabolism; 19, pigment metabolism; 20, phenylpropanoid metabolism; 21, lipid localization; 22, reproductive process in a multicellular organism; 23, chitin binding; 24, pattern binding; 25, chitinase activity; 26, polysaccharide binding; 27, response to chitin; 28, photosynthesis; 29, indolalkylamine metabolism; 30, tryptophan metabolism; 31, hormone metabolism; 32, regulation of hormone levels; 33, indole derivative metabolism; 34, indole and derivative metabolism; 35, response to cold; 36, response to bacterium; 37, auxin metabolism; 38, nucleosome assembly; 10, tetrapyrrole binding; 39, iron ion binding; 40, electron carrier activity; 9, heme binding; 8, monooxygenase activity; 41, oxygen binding; 42, RNA-dependent DNA replication; 43, anatomical structure homeostasis; 44, DNA metabolism; 45, adenyl ribonucleotide binding; 46, purine nucleoside binding; 47, ATP binding; 48, nucleotidyltransferase activity; 49, adenyl nucleotide binding; 50, RNA-directed DNA polymerase activity; 51, nucleoside binding; 52, purine ribonucleotide binding; 53, ribonucleotide binding; 54, DNA polymerase activity; 55, purine nucleotide binding; 56, nucleotide binding.

We are interested in differentially expressed genes between BT×623 and Keller. These include BT×623/Keller-specific genes (expressed only in BT×623/Keller), sucrose-regulated genes only in BT×623/Keller and genes with at least two times’ higher in BT×623/Keller. Based on our analysis (Figure 3), total of 4,015 probes were identified with differential expression between BT×623 and Keller. Among them, 3,436 genes with annotated loci were selected to investigate their functional divergence (Figure 4a). Firstly, we analyzed their amino acid sequence variations of these genes and found that total of 47% of differentially expressed genes showed no variation in their encoded protein sequences. The remaining genes were subjected to *Ka/Ks* analysis, which revealed that only 2.4% of these genes exhibited functional or protein divergence with *Ka/Ks* > 1 (Figure 4b). These data indicated that most of the differentially expressed genes between BT×623 and Keller were under purifying selection with functional constraints.

Since genes with either functional/protein (Figure 1d and e) or expression (Figure 4a) divergence may contribute to phenotypic variations (Figure 1a to c), we were interested in figuring out the difference in their functional annotation. Hence, we investigated Gene Ontology (GO) terms and identified overrepresented GO terms (Figure 4c-e). For each term, we identified GO-slim terms in three categories: molecular function (F), biological process (B), and cellular component (C) [15]. Our primary motivation was to evaluate whether these genes are biased toward particular functions. Our data showed that overrepresented BT×623-specific genes mainly functioned in apoptosis/cell death related biological functions or as helicase (pink column in Figure 4c) in molecular function. Keller-specific genes might play roles in reproductive cellular process and post-embryonic morphogenesis or with monooxygenase activity, heme and tetrapyrrole binding (blue column in Figure 4c). Interestingly, differentially expressed genes with at least twice higher in BT×623 also showed functions in apoptosis/cell death related biological processes (red column in Figure 4d). However, for the genes with twice higher expression in Keller, they showed overrepresented biological functions in multiple GO terms (black column in Figure 4d). Similarly, multiple GO terms have been identified with overrepresented biological functions or molecular functions for differentially expressed genes under sucrose treatment (Figure 4e). Some overlapping GO categories have been observed between sucrose regulated genes and these genes with expression only in Keller (Figure 4c and e). These categories include reproductive cellular process with item No. 6 for biological function and monooxygenase activity, heme and tetrapyrrole binding with No. 8, 9 and 10 for molecular function (blue numbers in Figure 4c and e). In addition, we also analyzed the genes with functional or protein divergence as indicated in Figure 1d. Similarly, multiple GO categories were detected with over-representation in both biological and molecular functions (Figure 4f). Among them, interestingly, genes with apoptosis/cell death related biological functions (as shown in Figure 4c and d) were also detected with over-representation (red numbers in Figure 4f).

### Promoter variation and expression divergence

**Figure 5:**
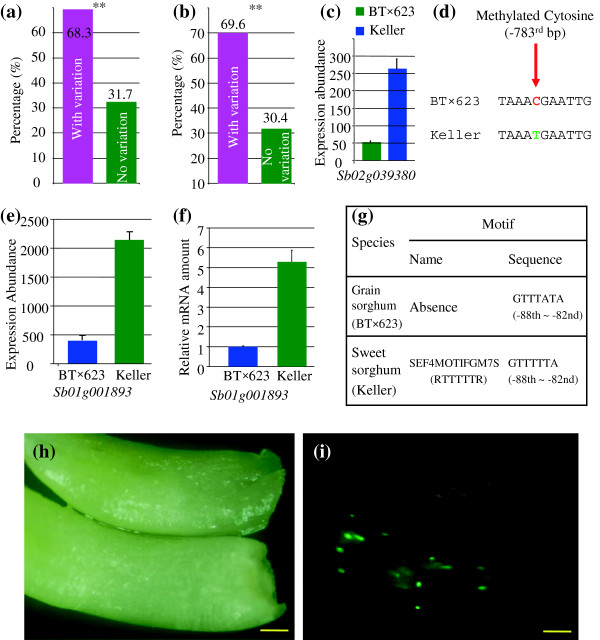
**Investigation of promoter variation and DNA methylation. (a)** Percentages of genes with and without variations in their promoter regions. **(b)** Percentages of promoter variation of genes with differential expression. **(c)** An example of a gene with the same promoter sequence but with differential expression abundance between BT×623 and Keller. **(d)** Differentially methylated cytosine in their promoter regions between BT×623 and Keller. **(e)** and **(f)** An example of a gene with differential expression abundance by microarray and qRT-PCR analysis, respectively. **(g)** Promoter motif analysis of the gene *Sb01g001893*. **(h)** and **(i)** Transient expression analysis of the promoter-GFP constructs. **(h)** The bright-field image under visible light. **(i**) The image showing GFP expression abundance under confocal microscopy in bombarded sorghum shoots. Top and bottom panels in **(h)** and **(i)** show the images from the promoters without motif SEF4MOTIFGM7S and with the motif, respectively. Bars in **(h)** and **(i)** are 250 μM. To test their transformation efficiency, total of 10 microscope views were selected in each cassette each repeat to count transformed cells with detectable GFP activity. On average, 20.2 and 19.9 cells in each view for the motif SEF4MOTIFGM7S-containing cassette and non motif cassette, were detected, respectively. No difference has been detected in their transformation efficiency by *t*-test (P<0.01).

To explore why considerable number of genes showed expression divergence, we examined their promoter variations. Among the total of 36,338 annotated genes, 68.3% of them showed variations in the 1.5 Kb promoter regions upstream of start codon of each gene and the remaining 31.7% of genes showed no promoter variation between BT×623 and Keller (Figure 5a). Among the differentially expressed genes, 69.6% of them showed promoter variation and 30.4% of them exhibited no variation in their 1.5 Kb promoter regions (Figure 5b). These data indicated that expression divergence was also observed in genes with identical promoter sequences but under different genome background. To explain the observation, we randomly selected 20 genes with identical promoter sequence in 1.5 Kb regions but with differential expression abundance between BT×623 and Keller for DNA methylation analysis. Our data showed that DNA methylation levels and patterns in promoter regions were different between these two varieties. One of the examples lies in the gene with locus name *Sb02g039380*. This gene showed significant difference in their expression abundance between BT×623 and Keller (Figure 5c). However, their 1.5 Kb promoter regions showed 100% homology. Bisulphate sequencing of these regions showed that they exhibited distinct differences in the percentages of methylation regions. For example, the cytosine at the -783^rd^ bp of the promoter in BT×623 was methylated whereas the corresponding locus was not methylated in Keller (Figure 5d). The result may provide an evidence to explain why the expression abundance in Keller was at least 4 times higher than that in BT×623.

Since up to 69.6% of promoters showed polymorphism between BT×623 and Keller, we also investigated how these variations contributed to the expression divergence. All the promoter sequences from differentially expressed genes were achieved from both BT×623 and Keller genomes and were then submitted to motif searches (see Methods). Over-represented motifs were identified according to their frequency presented in BT×623 and Keller (Additional files 2 and 3). For the genes expressed only in BT×623, one of the over-represented motifs is TATABOX3, which is critical for accurate transcription initiation. In Keller, some of the motifs have been mutated; as a result, no expression was detected. The motif HDZIP2ATATHB2 was over-represented in these genes with specific expression patterns or with higher expression level in BT×623. Matrix attachment regions (MARs) usually resulted in higher expression [16]. Over-represented MARTBOX motif may provide an evidence to explain why expression level in BT×623 is higher than that in Keller in corresponding genes. Both motifs SURE1STPAT21 and SURE2STPAT21 are sucrose responsive elements (SURE) and they are over-represented in the sucrose-regulated genes only in BT×623. For Keller-specific genes, the xylem-specific expression element was over-represented, suggesting that Keller might be different from BT×623 in xylem development. Among the sucrose-regulated genes only in Keller, the motif ABREZMRAB28 was over-represented, which functions in ABA and water-stress responses. This fact may imply the interaction of sucrose metabolism and abiotic stress signaling.

To further investigate how these variations in promoter motifs affect gene expression patterns, two promoters with difference in their motif structures between BT×623 and Keller were selected randomly from differentially expressed genes. Promoter-GFP cassettes were constructed and were then transferred to sorghum shoots for transient expression. One of the examples is based on the analysis of the gene with locus name *Sb01g001893*. This gene showed significant difference in their expression abundance between BT×623 and Keller by microarray (Figure 5e) and quantitative real time reverse transcription PCR (qRT-PCR, Figure 5f) analysis. Promoter sequence analysis showed that the motif SEF4MOTIFGM7S was absent in BT×623 but present in Keller (Figure 5g). The motif was within an enhancer and has been involved in the regulation of expression abundance [17,18]. We isolated the SEF4MOTIFGM7S-containing promoter from Keller. This promoter was mutated at the motif sequence from GTTTTTA to GTTTATA. Both promoters were used to drive GFP expression. The transient expression analysis showed that obviously stronger GFP signal were observed in the shoots where GFP was driven by the SEF4MOTIFGM7S-containing promoter (Figure 5h and i). These data suggested that SNP-mediated promoter motif modification might result in expression divergence.

### Gene duplication/expansion and expression divergence within and between BT×623 and Keller

**Figure 6:**
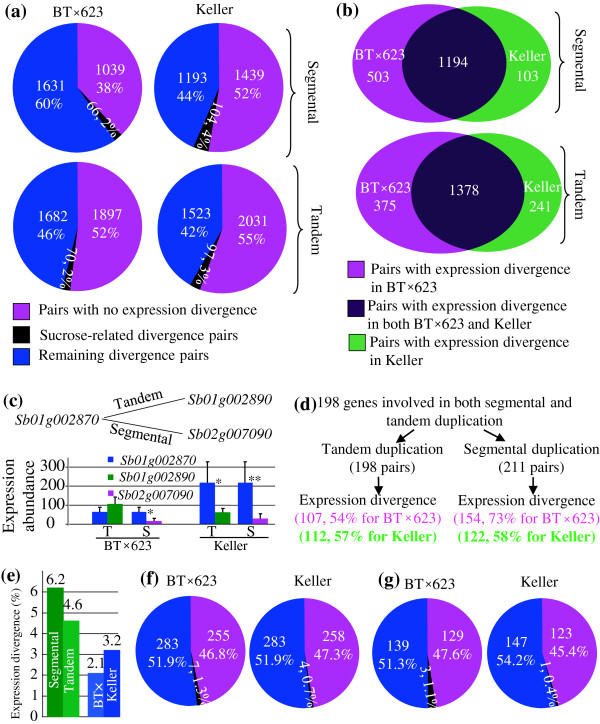
**Gene expansion and expression divergence within a species. (a)** The effect of segmental (top panel) and tandem (bottom panel) duplication on gene expression divergence. **(b)** Commonly or variety-specific expression divergence in BT×623 and Keller. **(c)** An example showing a gene undergoing both tandem and segmental duplication to give birth to new genes and their expression divergence. **(d)** Detected genes involved in both segmental and tandem duplication and their expression divergence. **(e)** The expression divergence between segmental and tandem duplication (green column) or between BT×623 and Keller (blue column) under sucrose treatment. **(f)** and **(g)** The effect of LTR-retrotransposons and CACTA elements on gene expression divergence. In **(f)** and **(g)**, pink color shows pairs with no expression divergence, black color shows sucrose-related divergence pair and blue color shows the remaining divergence pairs.

To explore the molecular basis of gene expression divergence within and between BT×623 and Keller, we further analyzed the effect of both gene duplication and mobile elements on expression divergence. We first examined the contribution of segmental and tandem duplication events to the expression divergence within BT×623 or Keller. After segmental duplication, up to 62% of duplicates including 2% of sucrose-related pairs exhibited expression divergence in BT×623 whereas only 48% of paralogs showed divergence in Keller (Figure 6a). For tandem duplication, 48% (including 2% of sucrose-related pairs) in BT×623 and 45% (including 3% of sucrose-related pairs) paralogs were detected with expression divergence (Figure 6a). Therefore, both varieties exhibited significant difference in expression divergence after segmental or tandem duplication (Z test, P<0.01). Majority of expression divergence was commonly detected in both BT×623 and Keller (Figure 6b). In BT×623, significantly higher percentage of segmentally duplicated genes showed expression divergence; however, in Keller, this percentage was reduced (Figure 6a). To further confirm the result obtained, genes involved in both segmental and tandem duplications were selected for further analysis. For example, the paralog of the gene *Sb01g002870* was supposed to be *Sb01g002890* by tandem duplication or *Sb02g007090* by segmental duplication (Figure 6c). In BT×623, only segmentally duplicated genes showed expression divergence; however, in Keller, two pairs of genes from both segmental and tandem duplications showed expression divergence (Figure 6c). Totally, we have detected 198 genes involved in this type of duplication. In BT×623, around 54% of tandemly duplicated pairs showed expression divergence whereas up to 73% of segmentally duplicated genes exhibited expression divergence in Keller, indicating the significant difference in their expression divergence (Figure 6d). However, in Keller, similar percentage of expression divergence was observed between segmental and tandem duplication (Figure 6d). A total of 6.2% of segmentally duplicated pairs showed expression divergence only under sucrose treatment, significantly higher than the percentage (4.6%) for tandem pairs (Z test, P<0.05, Figure 6e). Although lower expression divergence after segmental or tandem duplication was observed in Keller when compared with BT×623, higher percentage of duplicated pairs in Keller than in BT×623 showed expression divergence only under sucrose treatment. In Keller, up to 3.2% of duplicated pairs showed expression divergence only under sucrose treatment; in contrast, only 2.1% of pairs were observed in BT×623 (Figure 6e).

In addition to segmental and tandem duplication, we have also investigated the contribution of both LTR-retrotransposon and CACTA elements to expression divergence within BT×623 and Keller. Our data showed that more than 50% of expanded genes by these mobile elements were under expression divergence (Figure 6f and g), similar to the role of segmental/tandem duplication. However, only 11 and 4 pairs of expanded genes from LTR-retrotransposons and CACTA elements, respectively, showed expression divergence under sucrose treatment (Figure 6f and g). The data suggested the low contribution of mobile elements to sucrose related expression divergence either within BT×623 or Keller.

**Figure 7:**
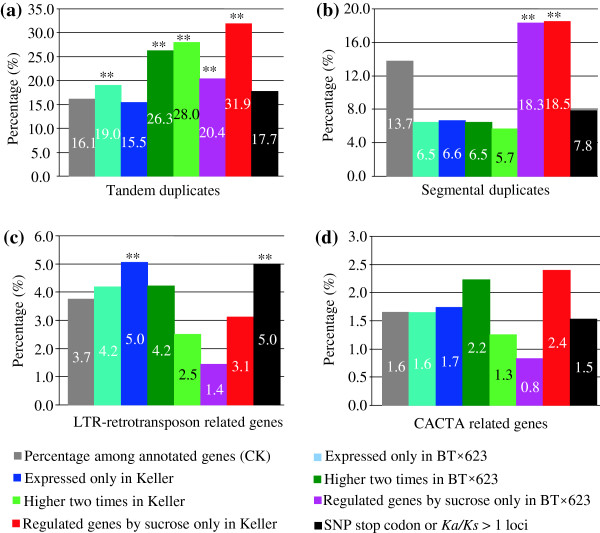
**Gene expansion and expression divergence between BT×623 and Keller. (a)** to **(d)** show the contribution rates of tandem duplication, segmental duplication, LTR-retrotransposon, CACTA mobile elements to gene expression divergence, respectively. The symbol “**” indicated the significant contribution of an expansion mode to gene expression divergence at P<0.01.

Subsequently, we were interested to analyze the mechanism of gene expression divergence between BT×623 and Keller. To explore the effect of gene duplication/expansion on expression divergence between these two varieties, we investigated the contribution of segmental/tandem duplication and mobile elements to the expression divergence. We first analyzed the contribution of tandem duplication to the expression divergence. Based on our analysis, tandemly duplicated genes accounted for 16.1% of total annotated sorghum genes. Analogically, 13.7%, 3.7% and 1.6% of total annotated sorghum genes were identified to be related to segmental duplication, LTR-retrotransposon and CACTA element mediated gene expansion, respectively (grey colored columns in Figure 7a-d). Among total divergent 765 genes with expression only in BT×623 (Figure 4a), 19.0% of them were evolved from tandem duplication (cyan colored column); the ratio is significantly higher (Z test, P<0.05) than the percentage of tandem duplicates among total annotated genes (CK, 16.1%, grey colored column in Figure 7a). Similarly, over-represented tandem genes were also observed in these genes with higher two times in BT×623 (26.3%, dark green colored column), higher two times in Keller (28.0%, light green colored column), regulation by sucrose only in BT×623 (20.4%, pink colored column), regulation by sucrose only in Keller (31.9%, red colored column), respectively (Figure 7a). For the genes expressed only in Keller or with SNP stop codon or with *Ka/Ks* >1, no significant difference was observed when compared with the control (brown colored column in Figure 7a). Subsequently, we examined the contribution of segmental duplication. We detected up to 18.3% genes regulated by sucrose only in BT×623 were involved in segmental duplication, statistically higher (Z test, P<0.01) than the percentage (13.7%) of segmental duplicates among total annotated genes. Overly-represented segmental genes (18.5%) were also observed in the genes regulated by sucrose only in Keller (Figure 7b). For the remaining types of differentially expressed genes, no significantly higher percentage of segmental genes was observed in our analysis. For LTR-retrotransposon related genes, over-represented genes were detected in these genes with expression only in Keller (5%) and with SNP stop codon or *Ka/Ks* > 1 (5%) (Figure 7c). However, for the CACTA-related genes, no significant difference was observed to contribute to differential expression between BT×623 and Keller (Figure 7d). Thus, our data suggested that both tandem and segmental duplication could have significantly contributed to gene expression divergence between BT×623 and Keller. Recently, Hollister et al., (2011) reported that transposable elements significantly contributed to expression divergence between *Arabidopsis thaliana* and *Arabidopsis lyrata*[19]. Thus, molecular and evolution basis of gene expression divergence in sorghum and Arabidopsis might be different.

## Discussion

### Expression divergence was genetically determined by functionally divergent genes between BT×623 and Keller

**Figure 8:**
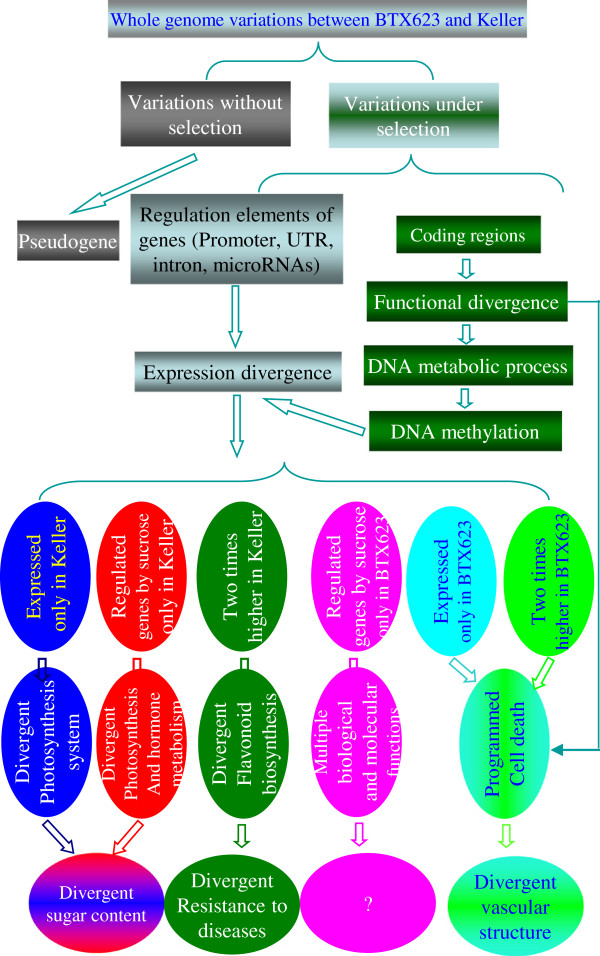
**Genome variation, expression divergence, phenotypic variation between BT×623 and Keller and their relationship.** Functionally significant genome variations between BT×623 and Keller might be subjected into selection pressure during evolution and variety domestication. Variations in gene coding regions might lead to gene functional divergence. One of the divergences was in genes involved in programmed cell death, which might lead to divergent vascular systems between BT×623 and Keller. Another divergence is in DNA methylation, which might contribute to gene expression divergence. Variations in promoters, UTRs, introns, microRNAs etc. might also result in expression divergence. BT×623 and Keller exhibited significant divergence in gene expression, which might contribute to divergent sugar content and disease resistance.

Genes with genetic variations might lead to pseudogenes without any selection pressure and then lose their functions. In contract, genes with sequence variations might evolve into new biological functions or expression patterns under certain selection pressures (Figure 8). Although large numbers of sequence variations have been detected, we have only detected 1,332 genes with functional or protein divergence between these BT×623 and Keller (Figure 1d to g). Around 40% of them showed no expression in either BT×623 or Keller and some of them should have been evolved into pseudogenes and others might be tissue-specific or stress-induced. Although functionally divergent genes between these two lines are limited, they might have played important roles in the divergence between these two varieties (Figure 8). Based on the over-represented genes involved in “biological process” in the Gene Ontology analysis, these genes might mainly be involved in programmed cell death and anatomical structure homeostasis (Figures 4f and 8). The analysis may imply that these genes function in anatomical divergence at the seedling stage. One of the observed differences between BT×623 and Keller is the developmental stage of tracheary elements during xylem formation (Figure 1b and c). BT×623 generally develops tracheary elements earlier than Keller. The development of tracheary elements are directly related to programmed cell death [20]. Thus, functionally divergent genes might contribute to the phenotypic divergence in xylem formation. Another over-represented biological function of divergent genes is involved in DNA metabolic process. This biological process is important for DNA methylation. In fact, among the functionally divergent genes, we have detected many genes directly related to DNA methylation, including these genes encoding various methyltransferases, helicases, histone deacetylases and histidine kinases and so on. These facts may imply the difference in DNA methylation between these two varieties, which were confirmed by our experiments (Figure 5). The difference in return regulates expression divergence between these two lines (Figure 8). Therefore, it is the genotypic divergence that leads to expression divergence.

Among 3436 annotated genes with differential expression between these two lines (Figure 4a), majority of them showed no functional divergence based on *Ka/Ks* analysis (Figure 4b). They were classified into 6 types of differentially expressed genes (Figures 7 and 8). Interestingly, over-represented genes with expression only in BT×623 or with two times higher of expression level in BT×623 showed similar Gene Ontology: programmed cell death (Figure 4c and d). This result might imply that not only genome variation but also expression divergence contribute to the difference in development of tracheary elements. Among differentially expressed genes with two times higher in BT×623, genes were much more enriched in their functions related to flavonoid biosynthesis (Figures 4d and 8). In sorghum and other plants, flavonoids play important roles in disease resistance [21,22]. Both BT×623 and Keller showed obvious difference in disease resistance. For example, anthracnose is one of the main diseases in sorghum. Keller is resistant to the disease [23] but BT×623 is susceptible [24]. The disease is flavonoid phytoalexin-dependent [25]. Among a total of 36,338 annotated genes, Liu et al. (2010) identified 6 favonoid structural genes encoding flavanone 3-hydroxylase, dihydroflavonol 4-reductase or anthocyanidin synthase [26]. Four of them, *Sb03g028880*, *Sb04g000260*, *Sb06g031790* and *Sb09g003710*, showed at least two times higher in their expression abundance in Keller than in BT×623. The result may provide an evidence to explain why Keller showed improved resistance to anthracnose. Thus, our data suggest that genes with two times higher in Keller might play a role in the divergence of disease resistance between BT×623 and Keller (Figure 8). On the other hand, over-represented molecular and biological functions of genes regulated by sucrose only in BT×623 are involved in multiple biological and metabolic processes (Figure 4e). Further study should be carried out to understand their roles in species divergence.

The remaining two sets of differentially expressed genes are those expressed only in Keller and regulated by sucrose only in Keller. Over-represented molecular functions of genes expressed only in Keller are monooxygenase activity, heme binding and tetrapyrrole binding (Figure 4c), which are required for tetrapyrrole biosynthesis. The biosynthesis pathway supplies important molecules for photosynthesis [27]. Thus, these two sets of genes mainly function in photosynthesis and hormone metabolism (Figures 4c, 4e, 8). More genes have been involved in this process in Keller than in BT×623, suggesting the differentiation of photosynthesis system between these two varieties. This result might provide an evidence to explain more sugar accumulation in sweet sorghum. However, differentiation of sugar accumulation between sweet and grain sorghum lines is complex and more components might have been involved in this process. Recently, Calvino et al. (2011) carried out the transcriptome characterization of small RNA component in the grain (BT×623) and sweet (Rio) sorghum stems [28]. Their data revealed that expression divergence of known miRNAs between BT×623 and Rio correlated with sugar content in their F2 population, suggesting a potential role of microRNA in stem sugar accumulation. The genes involved in sugar accumulated are not well characterized in sorghum due to the low heritability of the trait and its quantitative inheritance [28]. Our comparative analysis from re-sequencing data showed that genes with functionally divergent genes between BT×623 and Keller might not be directly involved in sugar accumulation. Interestingly, screening of sorghum genes linked to high sugar content indicated that 80% of differentially expressed genes between sweet and grain sorghum had their orthologs in rice suggesting limited contribution of their gene content for differentiating sorghum sugar accumulation [8,9]. Thus, our data and others [8,9,28] suggested that expression divergence should play important roles in the divergence of sugar accumulation between sweet and grain sorghum lines. In fact, changes of gene expression in eukaryotes often give rise to new phenotypes and the changes were frequently used as a proxy indicator of functional divergence of genes [29].

### Molecular basis and mechanisms of genome variations and expression divergence between BT×623 and Keller

Due to very limited genes with functional or protein divergence but with obvious phenotypic diversity between BT×623 and Keller, they provide an excellent and comparable materials to study the relationship between genotype and phenotype. Our re-sequencing data showed that majority of tandem and segmental duplications as well as transposition events of mobile elements occurred beyond the divergence between BT×623 and Keller. For duplication related gene expansion, we have analyzed both segmental and tandem duplications since both BT×623 and Keller showed similar whole genome duplication events. For transposition related gene expansion, we investigated only both LTR retrotransposons and CACTA super family since these two classes account for majority of mobile elements in sorghum [2]. We have investigated functional divergence of coding regions of expanded genes. Our data showed that less than 10% of expanded genes had evolved into new functions within a species (Figure 2c-f) or between BT×623 and Keller. We were wondering how these expanded genes could survive with only 10% of functional divergence. Subsequently, we investigated the expression divergence among these expanded genes. Interestingly, our results showed that around 50% of expanded genes showed expression divergence within BT×623 or Keller (Figure 6), suggesting that their expression divergence is a major mechanism to drive the retention of expanded genes by segmental/tandem duplication or transposition by mobile elements. On the other hand, among total of differentially expressed genes, tandem or segmentally duplicated genes were over-represented (Figure 7). This data suggested that expression divergence of expanded genes also significantly contributed to the divergence of two sorghum lines. Due to the significant contribution of expression divergence to phenotypic variation, we further analyzed the mechanism how these expanded genes were retained by expression divergence. Reasonably, our data showed that motif variations of promoter regions contributed to expression divergence (Figure 5). However, our data showed that expression divergence was also observed in genes with same promoter sequence. In this case, DNA methylation has been proven to play important roles in expression divergence between BT×623 and Keller. In fact, DNA methylation has been observed in sorghum and played a role in tissue-specific expression [30]. Our work demonstrated that differential DNA methylation might play a role in expression divergence not only within a genotype but also between closely related two genotypes. Since we analyzed expression patterns with only several samples under limited stress conditions, more expression divergence should be revealed within BT×623 / Keller or between these two varieties. Thus, higher expression divergence should be detected. However, thus an expression divergence is genetically determined by the divergence in genome level.

Substantial differences in gene expression patterns have been observed between closely related species [12-14]. Mechanisms underlying these differences have not yet fully understood. Generally, gene expression is regulated by various transcription factors. However, sequence divergence at transcription factor-binding sites accounts for only a small fraction of observed expression differences [13,31]. Our data also showed the limited expression divergence in genes encoding various transcription factors between BT×623 and Keller (Additional file 1a and b). Studies also suggested that chromatin regulators have a key role in generating expression diversity [13]. However, majority of the studies on gene expression divergence were carried out using closely related species (inter-species), little is known about the expression divergence within intra-species. Does a similar mechanism control the expression divergence? Interestingly, our data demonstrated that DNA methylation play important roles in gene expression divergence within intra-species (Figure 5a-d), indicating a similar mechanism to control the expression divergence within intra-species.

Another interesting issue is how the expression divergence leads to differential sugar accumulation between grain and sweet sorghum. Comparative expression analyses have been carried out in multiple species to investigate the differentially expressed genes under certain stress conditions between a pair of genotypes with obvious difference in a specific phenotypic trait. For example, between drought tolerant and sensitive rice cultivars, more drought up-regulated genes were detected in the sensitive than in the tolerant cultivars [32]. More salinity up-regulated genes were also identified in the salinity sensitive lines than in the tolerant lines [33]. Our data show a similar trend. More up-regulated genes by sucrose treatment in BT×623 were detected than that in Keller (Figure 3e and f). Based on the above mentioned data, we propose the hypothesis that higher sugar accumulation in Keller might be due to constitutive over-expression of these genes that function in sugar accumulation and were up-regulated in BT×623.

### Duplication and transposition and cultivar domestication

Sweet and grain sorghum lines have originated from the same subspecies and the former is a natural variant of the latter [3]. How sweet sorghum differs genetically from grain sorghum is not well characterized [34]. Modern sweet sorghum cultivars were developed from naturally variant sweet sorghum by sexual crossing and subsequent selection. What is the mechanism beyond the breeding selection? Plant genomes contain higher percentage of duplicated genes when compared with most of other eukaryotes [35]. Segmental, tandem duplications and transpositions by mobile elements have significantly contributed to such an event [2,36]. Both duplication and transposition provided basis for genetic variation. Expanded genes might give birth to new genes with divergent functions for better adaptability, competition for species existence. On the other hand, duplicate genes in Arabidopsis also exhibited higher percentages of expression divergence between or within species [37,38]. Similar results were observed in sorghum in this study. During artificial domestication of sweet sorghum, genetic variation was pyramided to contribute to the higher content of sugar accumulation by breeding program. Previous studies showed that tandem duplication played a role in the adaptive response to environmental stimuli [39]. Our data showed that tandem/segmental duplication played a role in the divergence of certain specific traits such as photosynthesis and disease resistance. As a result, tandem/segmental duplication might contribute to intra-species divergence and cultivar domestication under natural variation and artificial selection.

## Conclusions

The application of “next-generation sequencing” technique has greatly increased the speed and output of sequencing works with reduced costs. How to explain the phenotypic difference among closely related species is becoming more and more interesting since their genome sequencing data are more easily available. Our data showed that although both grain and sweet sorghum genomes exhibited considerable differences in their genome sequences, only limited divergence occurred in their functional genes. Thus, majority of phenotypic differences between these two varieties might not be directly due to the divergence of these functional genes. However, we have detected more than 3,000 differentially expressed genes between these two varieties. Such expression divergence was resulted from mutations in expression regulatory sequences and DNA methylation, which was genetically determined by functionally divergent genes between these two genomes. Further investigation showed that both tandem and segmental duplication played important roles in the genome evolution and expression divergence. Recently, Hollister et al. (2011) reported the contribution of transposable elements to gene expression divergence between *Arabidopsis thaliana* and *Arabidopsis lyrata*[19]. However, in sorghum, limited contribution of mobile elements to expression divergence has been observed, suggesting the difference in expression regulation between Arabidopsis and sorghum.

## Methods

### Plant materials, growth conditions, phenotypic investigation and sucrose treatments

Both grain and sweet sorghum (*Sorghum bicolor* L. Moench cultivars BT×623 and Keller were used for all experiments. They were planted in greenhouse and were grown under natural light and temperature conditions in Singapore. The detailed light conditions were listed in Additional file 4. The other climate conditions including temperature profiling were attached in Additional file 5. We have carried out 4 independent biological repeats. We planted sorghum lines on 5^th^ January 2010, 12^th^ January 2010, 5^th^ January 2011 and 12^th^ January 2011, respectively. They were planted in pots (18 cm depth, 25 cm top and 17 cm bottom diameters). Only one individual was planted in each pot. Soil used was from Singapore Far East Flora company (http://www.fareastflora.com). Brix degree was measured using a portable refractometer (N1 model, ATAGO, Japan). Since brix degrees are different in internodes, juice from the whole stalk was used for the test. In each biological repeat, total of 20 stalks were harvested at the stage of physiological maturity and were then immediately used for extracting juice followed by Brix degree test. Cryostat-microtome (CM3050S, Leica) was used for preparing slides for observing vascular system. The two-week old seedlings were collected and washed using fresh water with minimum root damage. They were then subjected to 5% of sucrose solution for treatment. Samples were then collected in 0, 2, and 6 h intervals, respectively for microarray analysis as shown below.

### Custom microarray design, hybridization and data analysis

Custom 60-mer oligonucleotide probes were designed using the publicly available eArray software (Agilent Technologies). In sorghum, total of 36,338 coding regions were annotated (http://www.phytozome.net/sorghum), from which 35,577 probes were designed and the remaining around 1,000 genes were not suitable for probe design due to the sequence homology to other genes. We have also collected total of 209,835 ESTs from the NCBI EST database (http://www.ncbi.nlm.nih.gov/dbEST/). We used these sequences for BLAST searches against all annotated genes and we found 6,400 non-abundant ESTs with no homology to these annotated genes. All these ESTs were also used for probe design. Therefore, we have designed total of 41,977 probes for microarray analysis (Additional file 6). These probe sequences were then submitted for manufacturing on 4×44k format of chips. The expression analysis was carried out using 14-day old whole seedlings from both grain and sweet sorghum lines under sucrose treatment (Additional file 7a). Two biological replicates were carried out for both control and sucrose treatments, resulting in a dataset of 12 microarrays. Total RNA quality was analyzed by nanodrop reading and Agilent Bioanalyzer running (Additional file 7b). The data quality was assessed by measuring the correlation coefficients (Additional file 7c). These analyses suggested the high quality of the data obtained in this study. On the other hand, we have also validated our data by quantitative real-time reverse transcription polymerase chain reaction (qRT-PCR) (Additional file 7d).

### Determination of over-represented genes and their GO categories

GO assignments for sorghum genes were obtained from the DOE Joint Genome Institute database (http://www.jgi.doe.gov/). Three top GO categories including biological processes, molecular functions and cellular components [40] were analyzed. Gene Set Enrichment Analysis (GSEA, [41]) was used to determine over-represented genes and their GO categories. To test if tandem/segmental duplication or transposition by mobile elements significantly contributes to expression divergence between BT×623 and Keller or within these species, the observed percentage of each type of duplicates with expression divergence (y1) among total duplicates from this type (n1) was compared with the expected percentage by statistic analysis. The expected percentage was estimated from the number of each type of differentially expressed genes (y2) among total annotated genes (n2). The *u* value was calculated using the formula as shown below and the value was converted to P value, which was used to estimate the statistic significance. $u=\left({\overset{⌢}{p}}_{1}-{\overset{⌢}{p}}_{2}\right)/\sigma_{{\overset{⌢}{p}}_{1}-{\overset{⌢}{p}}_{2}},\mathrm{where}\;{\overset{⌢}{p}}_{1}=y1/n1\;{\overset{⌢}{p}}_{2}=y2/n2,\text{and}\;\sigma_{{\overset{⌢}{p}}_{1}-{\overset{⌢}{p}}_{2}}=\sqrt{\left(\frac{y1+y2}{n1+n2}\right)\left(1-\frac{y1+y2}{n1+n2}\right)\left(\frac{1}{n1}+\frac{1}{n2}\right)}$

### Expression confirmation of sorghum genes by qRT-PCRs

Microarray expression data were confirmed by qRT-PCR. Total of 48 genes were randomly selected for the analysis. All gene-specific primers were designed by Applied Biosystems Primer Express® software. The amplification of a *SbUBQ5* gene was used as an internal control to normalize the data. All primer sequences were listed in Additional file 8. The qRT-PCR analyses were carried out using the AB power SYBR Green PCR Master mix kit (Applied Biosystems) according to the manufacturer’s protocol. The threshold cycle (C_T_) value was automatically calculated based on the changes in fluorescence of SYBR Green I dye in every cycle monitored by the ABI 7900 system software. The mRNA relative amount was used to evaluate gene expression level as 2– ^ΔΔCT^. Here, ΔC_T_ = C_T target_ – C_T__reference_ and ΔΔC_T_ = ΔC_T__test__sample_ – ΔC_T__calibrator__sample_. The ΔC_T_ values were used for *t*-test, which will yield an estimation of ΔΔC_T_.

### Detection of promoter motifs and DNA methylation by bisulfite sequencing

Promoter sequences 1Kb upstream of start codon of each gene were retrieved from both BT×623 and Keller genomes. Promoter motifs were detected by submitting these promoter sequences to the PLACE database [42]. A small program was designed to investigate the frequency of a promoter motif. Over-represented motifs were detected by comparing percentages *via u* test. For detection of DNA methylation, genomic DNA samples from BT×623 and Keller were subjected to sodium bisulphite treatment using the EpiXplore Methyl detection Kit (Clontech) following the procedures recommended by the manufacture. The Methyl Primer Express® Software v1.0 (Applied Biosystems, USA) was used for the design of bisulphite primers as listed in Additional file 8. PCR products were cloned into pGEM-T Easy vector for sequencing.

### Functional characterization of promoter motifs by promoter-GFP transient expression analysis

An around 1Kb of sorghum promoter from the ATG start codon was amplified from the Keller genomic DNA by PCR amplification using the primers as listed in Additional file 8. After verification by sequencing, the fragment was cloned in front of GFP reporter gene, and then subcloned into pCAMBIA1300 Ti-derived binary vector. The new binary vector was named as Keller_motif_GFP vector. The site-directed mutagenesis PCR was carried out using another set of primers as listed in Additional file 8 to produce the motif presented in the BT×623 genome. The mutated vector was named as BT×_motif_GFP vector. Both vectors were then introduced into both BT×623 and Keller shoot cells by the Biolistic PDS-1000/He particle delivery system (Bio-Rad). GFP activity and intensity were visualized and analyzed under a confocal microscope (Zeiss, Germany).

### Sorghum databases and annotation

The Keller genome re-sequencing data were downloaded from the sweet and grain sorghums database (http://gigadb.org/sweet-and-grain-sorghums/). The sequences and annotation of the sorghum genome BT×623 were achieved from the sorghum genome database (http://www.phytozome.net/sorghum).

### Identification of genes encoding transcription factors and carbohydrate metabolism related enzymes

Total of 84 families of transcription factors [43] were selected for genome-wide identification. The GRASSIUS database was also used for the identification of sorghum transcription factors [44]. For identification of genes encoding carbohydrate metabolism related enzymes, metabolism pathways were identified according to the description in the KEGG database ( [45]; http://www.genome.jp/kegg/pathway.html). For each family of transcription factors or carbohydrate enzymes, conserved motifs or domains were retrieved using the GenomeNet Database Resources (http://www.genome.jp/). These motif or domain sequences from seed members were obtained from the Pfam database (http://pfam.sanger.ac.uk/). Their seed amino acid sequences were used to construct Hidden Markov Model (HMM) profiles using HMMER 2.3.2 (http://hmmer.janelia.org/). Using the profile HMMs, we scanned the sorghum annotated protein database and to search for all putative transcription factors and carbohydrate metabolism related enzymes. These members were confirmed by further motif and domain analysis. All identified genes encoding transcription factors and carbohydrate metabolism related enzymes were listed in Additional files 9 and 10, respectively.

### Genome-wide identification of tandem and segmental duplication

Tandemly duplicated sorghum genes were determined using annotated sorghum proteins downloaded from the Phytozome sorghum genome database (http://www.phytozome.net/sorghum). All proteins were screened in an all-versus-all BLAST searches using BLOSUM62 matrix with an E value of less than 0.01. Pairs of matching peptides were identified with 70% or higher sequence identity and with a minimum of 30% of the query length. Pairs of matching proteins were clustered into families using a transitive closure algorithm (if A = B and B = C, then A = C). Tandemly duplicated genes were scored when two genes from the same family were located on the same chromosome, and were separated by a maximum 10 unrelated genes. The information for sorghum gene segmental duplication was obtained from the plant genome duplication database (http://chibba.pgml.uga.edu/duplication/). Both tandemly and segmentally duplicated genes were listed in Additional files 11 and 12, respectively.

### Genome-wide detection of mobile elements and related genes

We executed the LTR_Finder program [46] to genome-widely identify full-length sorghum LTR retrotransposons. The result was presented in Additional file 13. To identify CACTA DNA transposon elements, we first carried out BLASTN searches using both terminal inverted repeats (TIRs) and subterminal repeats (TRs) of the elements. The TIRs and TRs were from multiple species [47-49]. These searches generated two sets of BLAST hits (E ≤ 1^e-5^) including 5’ and 3’ terminal regions. Full-length CACTA elements were identified by matching two terminal regions with less than 30,000 bp in length. We also built a HMM profile using HMMER 2.3.2 with default values. Seed TIRs and TRs were selected from multiple species. Using the profile HMMs, we scanned the sorghum genome sequences and looked for hits that were separated by at least 200 bp and that were no more than 30,000 bp in the proper orientation. We then manually inspected these putative CACTA elements to remove any remaining artifacts by comparing their target site duplication/TIR sequences. The identified CACTA elements were presented in Additional file 14. These genes fully or partially located within a mobile element were regarded as LTR/CACTA-related genes and were listed in Additional files 13 and 14, respectively.

### Estimation of *Ka/Ks* ratios and C-value test

For *Ka* and *Ks* estimation, amino acid sequences were aligned and were subsequently transferred to the original cDNA sequences using the PAL2NAL program [50]. Pairs were selected while a minimum 70% of queried protein coding regions were aligned with an E value threshold at 10^-8^. Both *Ka* and *Ks* values were then estimated using the yn00 program of the PAML4b package [51]. The *Ka/Ks* ratios were also used for evaluating the functional divergence by the C value test [52].

### Accession numbers

All expression data have been deposited into the NCBI GEO database (http://www.ncbi.nlm.nih.gov/geo) under the serial number GSE36689.

## Abbreviations

EST: Expressed sequence tag; Indel: Insertions/deletion; HMM: Hidden Markov Model; Ka: Nonsynonymous substitutions per site; Ks: Synonymous substitutions per site; LTR: Long terminated direct repeat; MARs: Matrix attachment regions; MRCA: Most recent common ancestor; qRT-PCR: Quantitive real time reverse transcription PCR; SNP: Single nucleotide polymorphism; SURE: Sucrose responsive elements; SV: Structural variation; TRs: Subterminal repeats; TSD: Target-site duplications.

## Competing interests

The authors declare that they have no competing interests.

## Authors’ contributions

SR supervised the study. SYJ conceived of the study and carried out most of the work. ZM performed the promoter-GFP transient expression analysis and DNA methylation by bisulfite sequencing. JV carried out qRT-PCR. All authors read and approved the final manuscript.

## Supplementary Material

Additional file 1 Differentially expressed genes encoding transcription factors or carbohydrate metabolism related genes. Click here for file

Additional file 2 Over-represented promoter motifs in BT×623. Click here for file

Additional file 3 Over-represented promoter motifs in Keller. Click here for file

Additional file 4 Singapore day light conditions in years 2010 and 2011 (two sheets). Click here for file

Additional file 5 Singapore climate graph. Click here for file

Additional file 6 Probe designing for custom microarray chips. Click here for file

Additional file 7 Sampling and quality assessment of the Microarray expression data. Click here for file

Additional file 8 Primer sequences used for qRT-PCR and detection of DNA methylation. Click here for file

Additional file 9 Genome-wide identification of genes encoding transcription factors in sorghum. Click here for file

Additional file 10 Genome-wide identification of genes encoding carbohydrate metabolism related enzymes in sorghum. Click here for file

Additional file 11 Tandemly duplicated genes in sorghum. Click here for file

Additional file 12 Segmentally duplicated genes in sorghum. Click here for file

Additional file 13 Genome-wide identification of full-length LTR-retrotransposons and related genes. Click here for file

Additional file 14 Genome-wide identification of CACTA elements and related genes. Click here for file
