# Supplementary material for: Genetic variation and expression diversity between grain and sweet sorghum lines
Source: BMC Genomics. 2013 Jan 16;14:18. doi: 10.1186/1471-2164-14-18 (PMC3616923; doi:10.1186/1471-2164-14-18)
Supplement: Additional file 1 — Differentially expressed genes encoding transcription factors or carbohydrate metabolism related genes. [file 1471-2164-14-18-S1.ppt]

## Slide 1
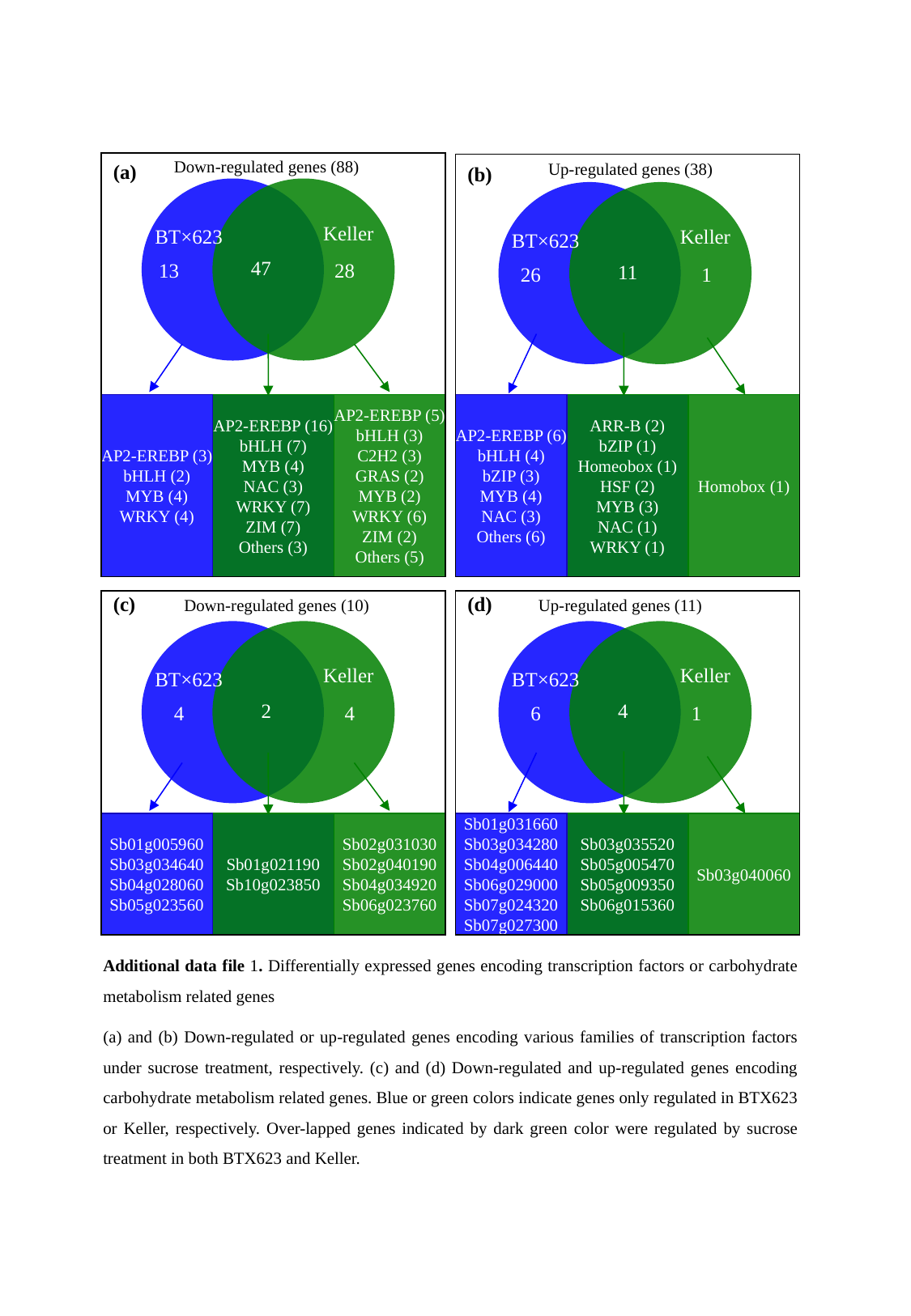

Down-regulated genes (88)
Up-regulated genes (38)
(a)
(b)
Keller
BT×623
47
13
28
Keller
BT×623
11
26
1
AP2-EREBP (3)
bHLH (2)
MYB (4)
WRKY (4)
AP2-EREBP (16)
bHLH (7)
MYB (4)
NAC (3)
WRKY (7)
ZIM (7)
Others (3)
AP2-EREBP (5)
bHLH (3)
C2H2 (3)
GRAS (2)
MYB (2)
WRKY (6)
ZIM (2)
Others (5)
AP2-EREBP (6)
bHLH (4)
bZIP (3)
MYB (4)
NAC (3)
Others (6)
ARR-B (2)
bZIP (1)
Homeobox (1)
HSF (2)
MYB (3)
NAC (1)
WRKY (1)
Homobox (1)
(c)
(d)
Down-regulated genes (10)
Up-regulated genes (11)
Keller
BT×623
 2
 4
 4
Keller
BT×623
4
6
1
Sb01g005960
Sb03g034640
Sb04g028060
Sb05g023560
Sb01g021190
Sb10g023850
Sb02g031030
Sb02g040190
Sb04g034920
Sb06g023760
Sb01g031660
Sb03g034280
Sb04g006440
Sb06g029000
Sb07g024320
Sb07g027300
Sb03g035520
Sb05g005470
Sb05g009350
Sb06g015360
Sb03g040060
Additional data file 1. Differentially expressed genes encoding transcription factors or carbohydrate metabolism related genes
(a) and (b) Down-regulated or up-regulated genes encoding various families of transcription factors under sucrose treatment, respectively. (c) and (d) Down-regulated and up-regulated genes encoding carbohydrate metabolism related genes. Blue or green colors indicate genes only regulated in BTX623 or Keller, respectively. Over-lapped genes indicated by dark green color were regulated by sucrose treatment in both BTX623 and Keller.
