# Supplementary material for: Genetic variation and expression diversity between grain and sweet sorghum lines
Source: BMC Genomics. 2013 Jan 16;14:18. doi: 10.1186/1471-2164-14-18 (PMC3616923; doi:10.1186/1471-2164-14-18)
Supplement: Additional file 2 — Over-represented promoter motifs in BT×623. [file 1471-2164-14-18-S2.doc]

**Additional data file 2.** Over-represented promoter motifs in BT×623

| Gene expression patterns | Motif name | Description |
| --- | --- | --- |
| Expressed only in BT×623 | TATABOX3 | Critical for accurate transcription initiation |
|  | HDZIP2ATATHB2 | Regulated by light signals and as a negative autoregulator of its own gene |
| Higher two times in BT×623 | HDZIP2ATATHB2 | Regulated by light signals and as a negative autoregulator of its own gene |
|  | MARTBOX | Motif found in matrix attachment region, MAR |
| Regulated genes by sucrose only in BT×623 | SURE1STPAT21 and SURE2STPAT21 | Sucrose Responsive Element (SURE) |
|  | IBOXCORE | Conserved sequence upstream of light-regulated genes |
