# Supplementary material for: Genetic variation and expression diversity between grain and sweet sorghum lines
Source: BMC Genomics. 2013 Jan 16;14:18. doi: 10.1186/1471-2164-14-18 (PMC3616923; doi:10.1186/1471-2164-14-18)
Supplement: Additional file 3 — Over-represented promoter motifs in Keller. [file 1471-2164-14-18-S3.doc]

**Additional data file 3.** Over-represented promoter motifs in Keller

| Gene expression patterns | Motif name | Description |
| --- | --- | --- |
| Expressed only in Keller | VSF1PVGRP18 | Xylem-specific expression element |
|  | CDA1ATCAB2 | Dark response element of chlorophyll a/b-binding protein2 gene |
| Higher two times in Keller | ELRENTCHN50 | Elicitor-responsive element |
| Regulated genes by sucrose only in Keller | ABREZMRAB28 | ABA and water-stress responses |
|  | AT1BOX | Promoter elements of the genes for tobacco chlorophyll a/b binding proteins |
