# Supplementary material for: Genetic variation and expression diversity between grain and sweet sorghum lines
Source: BMC Genomics. 2013 Jan 16;14:18. doi: 10.1186/1471-2164-14-18 (PMC3616923; doi:10.1186/1471-2164-14-18)
Supplement: Additional file 5 — Singapore climate graph. [file 1471-2164-14-18-S5.ppt]

## Slide 1
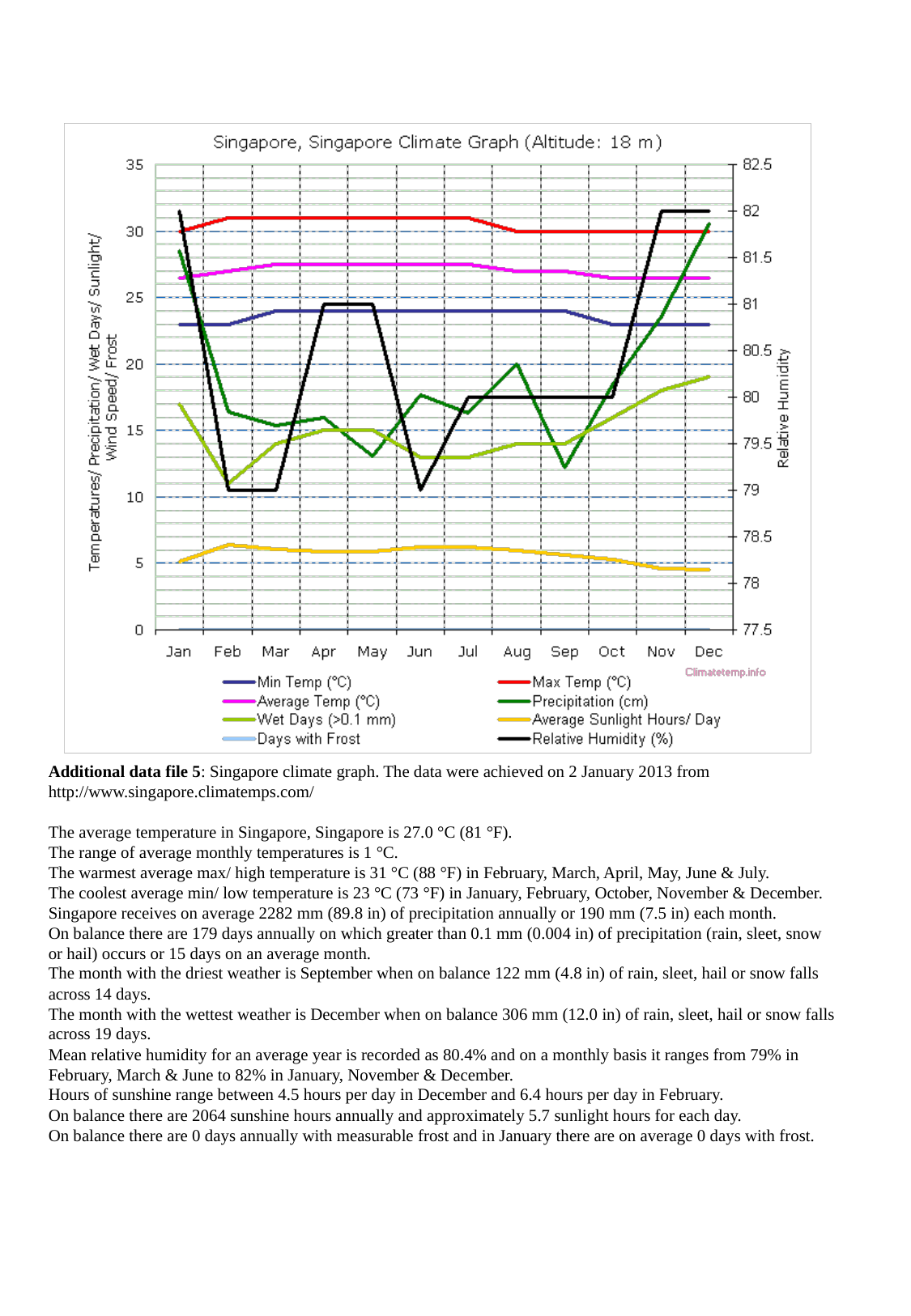

Additional data file 5: Singapore climate graph. The data were achieved on 2 January 2013 from http://www.singapore.climatemps.com/
The average temperature in Singapore, Singapore is 27.0 °C (81 °F).
The range of average monthly temperatures is 1 °C.
The warmest average max/ high temperature is 31 °C (88 °F) in February, March, April, May, June & July.
The coolest average min/ low temperature is 23 °C (73 °F) in January, February, October, November & December.
Singapore receives on average 2282 mm (89.8 in) of precipitation annually or 190 mm (7.5 in) each month.
On balance there are 179 days annually on which greater than 0.1 mm (0.004 in) of precipitation (rain, sleet, snow or hail) occurs or 15 days on an average month.
The month with the driest weather is September when on balance 122 mm (4.8 in) of rain, sleet, hail or snow falls across 14 days.
The month with the wettest weather is December when on balance 306 mm (12.0 in) of rain, sleet, hail or snow falls across 19 days.
Mean relative humidity for an average year is recorded as 80.4% and on a monthly basis it ranges from 79% in February, March & June to 82% in January, November & December.
Hours of sunshine range between 4.5 hours per day in December and 6.4 hours per day in February.
On balance there are 2064 sunshine hours annually and approximately 5.7 sunlight hours for each day.
On balance there are 0 days annually with measurable frost and in January there are on average 0 days with frost.
