# Supplementary material for: Genetic variation and expression diversity between grain and sweet sorghum lines
Source: BMC Genomics. 2013 Jan 16;14:18. doi: 10.1186/1471-2164-14-18 (PMC3616923; doi:10.1186/1471-2164-14-18)
Supplement: Additional file 6 — Probe designing for custom microarray chips. [file 1471-2164-14-18-S6.ppt]

## Slide 1
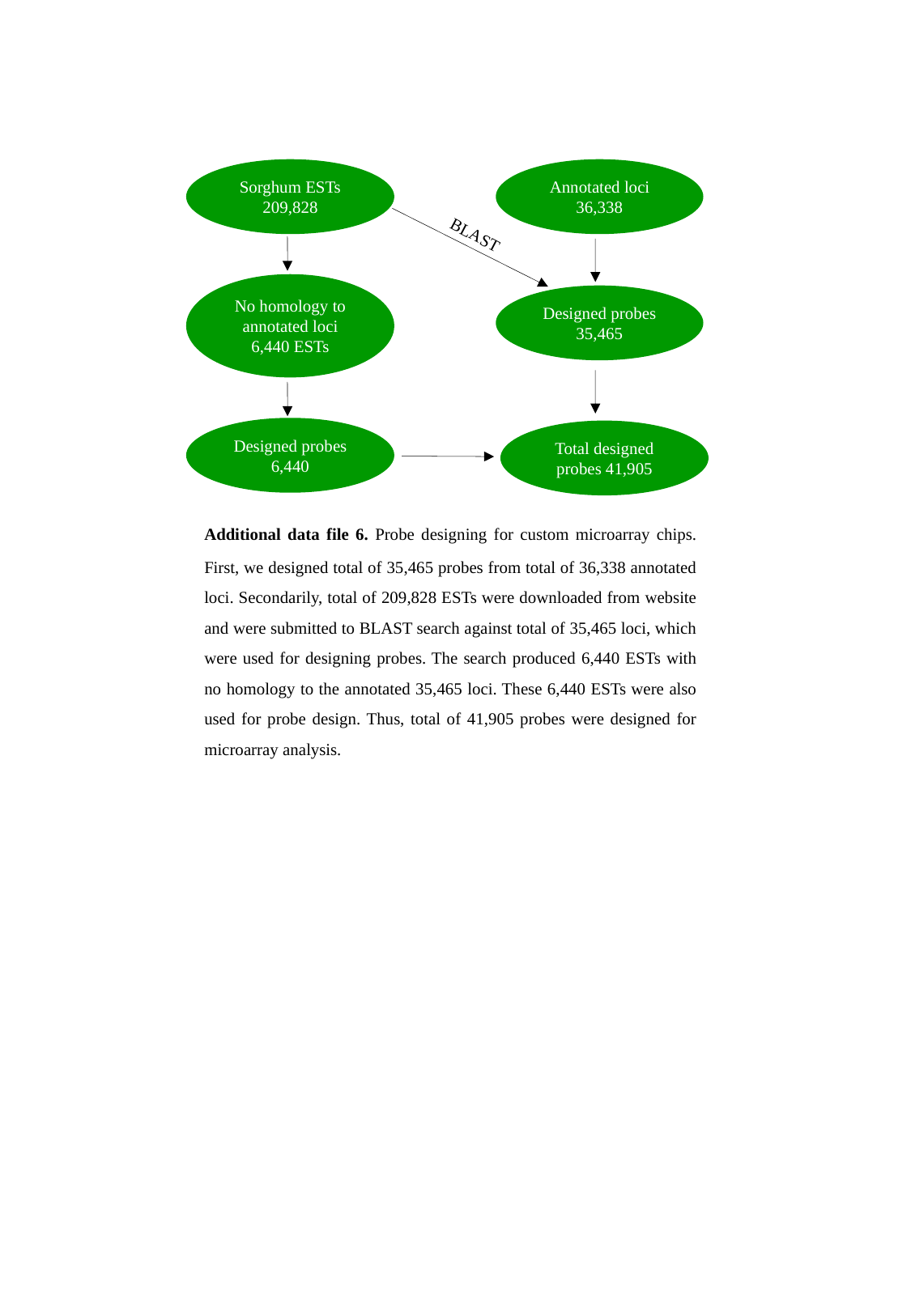

Sorghum ESTs 209,828
Annotated loci 36,338
BLAST
No homology to annotated loci 6,440 ESTs
Designed probes 35,465
Designed probes 6,440
Total designed probes 41,905
Additional data file 6. Probe designing for custom microarray chips. First, we designed total of 35,465 probes from total of 36,338 annotated loci. Secondarily, total of 209,828 ESTs were downloaded from website and were submitted to BLAST search against total of 35,465 loci, which were used for designing probes. The search produced 6,440 ESTs with no homology to the annotated 35,465 loci. These 6,440 ESTs were also used for probe design. Thus, total of 41,905 probes were designed for microarray analysis.
