# Supplementary material for: Genetic variation and expression diversity between grain and sweet sorghum lines
Source: BMC Genomics. 2013 Jan 16;14:18. doi: 10.1186/1471-2164-14-18 (PMC3616923; doi:10.1186/1471-2164-14-18)
Supplement: Additional file 7 — Sampling and quality assessment of the Microarray expression data. [file 1471-2164-14-18-S7.ppt]

## Slide 1
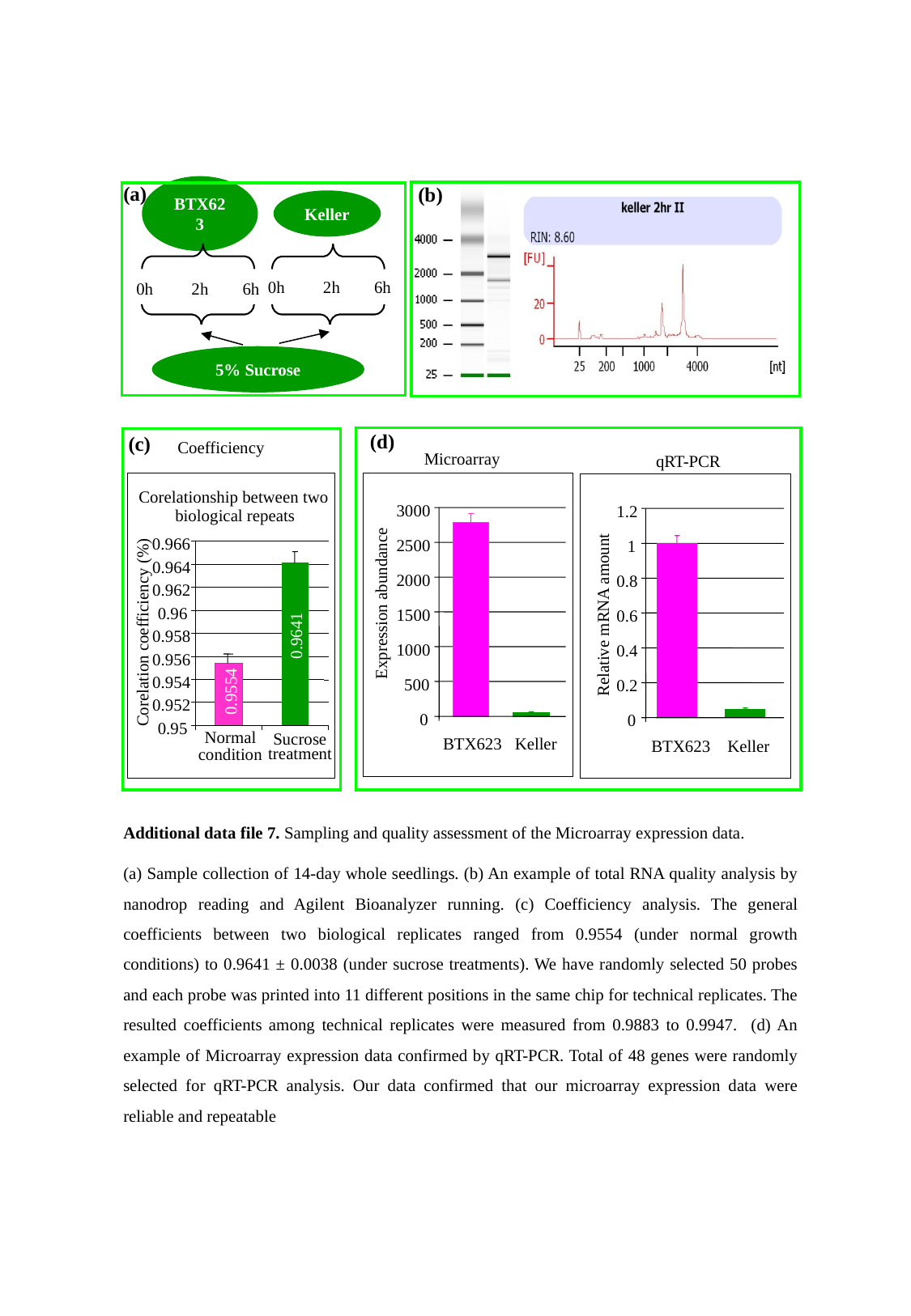

(a)
(b)
BTX623
Keller
0h 2h 6h
0h 2h 6h
5% Sucrose
(d)
(c)
Coefficiency
Microarray
qRT-PCR
Corelationship between two
biological repeats
0.966
0.964
0.962
0.96
0.958
0.956
0.954
0.952
0.95
0.9641
Corelation coefficiency (%)
0.9554
Normal
Sucrose
treatment
condition
3000
1.2
2500
1
2000
0.8
Expression abundance
1500
Relative mRNA amount
0.6
1000
0.4
500
0.2
0
0
BTX623 Keller
BTX623 Keller
Additional data file 7. Sampling and quality assessment of the Microarray expression data.
(a) Sample collection of 14-day whole seedlings. (b) An example of total RNA quality analysis by nanodrop reading and Agilent Bioanalyzer running. (c) Coefficiency analysis. The general coefficients between two biological replicates ranged from 0.9554 (under normal growth conditions) to 0.9641 ± 0.0038 (under sucrose treatments). We have randomly selected 50 probes and each probe was printed into 11 different positions in the same chip for technical replicates. The resulted coefficients among technical replicates were measured from 0.9883 to 0.9947. (d) An example of Microarray expression data confirmed by qRT-PCR. Total of 48 genes were randomly selected for qRT-PCR analysis. Our data confirmed that our microarray expression data were reliable and repeatable
